# Estimating, monitoring, and forecasting COVID-19 epidemics: a spatiotemporal approach applied to NYC data

**DOI:** 10.1038/s41598-021-88281-w

**Published:** 2021-04-27

**Authors:** Vinicius V. L. Albani, Roberto M. Velho, Jorge P. Zubelli

**Affiliations:** 1 Universidade Federal de Santa Catarina, Campus Trindade, 88, Florianopolis, SC 040-900 Brazil; 2 Federal University of Rio Grande do Sul, PO Box 15064, Av. Bento Gonçalves, 9500, Porto Alegre, RS 91501-970 Brazil; 3 Khalifa University of Science and Technology, PO Box 127788, Abu Dhabi, United Arab Emirates

**Keywords:** Applied mathematics, Nonlinear phenomena

## Abstract

We propose a susceptible-exposed-infective-recovered-type (SEIR-type) meta-population model to simulate and monitor the (COVID-19) epidemic evolution. The basic model consists of seven categories, namely, susceptible (S), exposed (E), three infective classes, recovered (R), and deceased (D). We define these categories for n age and sex groups in m different spatial locations. Therefore, the resulting model contains all epidemiological classes for each age group, sex, and location. The mixing between them is accomplished by means of time-dependent infection rate matrices. The model is calibrated with the curve of daily new infections in New York City and its boroughs, including census data, and the proportions of infections, hospitalizations, and deaths for each age range. We finally obtain a model that matches the reported curves and predicts accurate infection information for different locations and age classes.

## Introduction

During December 2019, in Wuhan, China, many cases of severe acute respiratory syndrome, caused by an unknown virus, were registered. Since then, the virus was named SARS-CoV-2, and the corresponding disease was designated by the acronym COVID-19, which means coronavirus disease 2019. On 11-Mar-2020, a pandemic was declared by the World Health Organization (WHO). As of 20-Feb-2021, SARS-Cov-2 has infected more than 111 million individuals and has caused more than 2.46 million deaths worldwide.

One clear message from the above history of the pandemic so far is that the study and management of the crisis calls for the use of spatiotemporal epidemiological models and their appropriate calibration using mathematical tools from numerical analysis and regularization theory^1,2^. This is the first goal of the present article.

The mathematical modeling of complex phenomena such as the COVID-19 outbreak involves building on previous models adopted in the literature^3–7^. It also involves considering specificities associated to the current outbreak. Here, the insights from social and behavioral science can considerably help the development of realistic models^8^. One natural conclusion is that the networking aspects of the epidemiological spread related to human interaction in urban areas are important. These aspects are included in our model through the construction of the spatial network and the age and sex interactions^9^.

It is now well documented that the likelihood of developing the more severe form of the disease increases dramatically with age^10–14^. In addition, the infection appears to be more frequent in older people^14^. These observations indicate that age plays an important role in possible containment and mitigation measures. Sex also appears to play a major role in COVID-19 outcomes, as documented in^15,16^. The sex dependency of COVID-19 may be linked, for example, to the differences in the behavior of male and female individuals, such as in the use of masks or in social distancing^8^. Incorporating age and sex structure in the model is the second goal of this article.

The absence of a universally available vaccine and of an effective treatment, at the time of writing of this article, considerably limits the number of possible actions to control the spread of the disease and the subsequent volume of hospitalizations and deaths. Thus, only containment or mitigation policies, such as quarantine or lockdown, may be applied. However, such measures have been causing an unprecedented impact on the economy and labor market, leading to massive unemployment and recession^17^. The International Monetary Fund revised its forecast in April 2020, predicting a 4.9% drop in global output in 2020^18^.

Quantifying and tracking the disease spread in different places and age ranges, as well as its impact on the health system, is useful for deciding whether the lockdown measures can be relaxed, gradually allowing the return of economic activity. This is the third goal of this work.

Moreover, an accurate forecasting of the number of regular hospital and intensive care unit (ICU) beds enables better use of public resources, bringing economic relief.

Susceptible-Exposed-Infected-Recovered (SEIR) and Susceptible-Infected-Recovered (SIR) models have been used to describe different disease outbreaks dynamics since the seminal work reported in^19^. See also^20^ in a textbook format. Recently, several studies^11,21–26^ have applied SIR- and SEIR-type models, or their extensions including fractional derivatives^3–7^, to describe the COVID-19 epidemic, including different features, such as geographical information and time-dependent transmission parameters.

In this article, we also propose a versatile SEIR-type model applied to COVID-19 epidemic dynamics. Our model takes into consideration different levels of disease severity, its impact on age ranges, and the distribution of the population in different locations. Following^10^, individuals in a severe state of this disease are considered in our model as hospitalized, while those critically ill are considered to be in an ICU. The interactions between classes of infected and susceptible individuals from different age-ranges, sexes, and places are defined by time-dependent transmission matrices. If appropriately calibrated with up-to-date data on daily new infections, such matrices can be used to reconstruct the status of the disease spread and allow us to verify the impact of containment measures.

Regarding vaccines, the flexible general form of our model can be used to design vaccination strategies that account for age, sex, and spatial distribution of susceptible population. From the sanitary point of view, such designed strategies may break the transmission chain of the disease while optimizing the costs of immunization of the population on the financial side.

The dependence of disease severity on age-range and sex is translated into the model through transmission rates and the rates of recovery, hospitalization, ICU admission, and death. The values of these rates are based on publicly available datasets and recent studies that analyzed, among other characteristics, the relationship between COVID-19 severity and age^10,14,26,27^ and sex^15,16^. Other parameters, such as mean incubation time and case fatality rate outside ICU, were obtained from^12,13,28–31^.

As mentioned above, our proposed model also accounts for spatial information. Policy makers can then identify clusters of uncontained disease spread in real time, isolate them, and later verify if the chosen imposed restrictions measures were effective. Moreover, the model can be used to detect which regions should be reopened first, thus reducing the economic impact of a lockdown. Once an effective vaccine is available, the model can be used to target regions where there are clusters of infected individuals. This approach will then be used to create immunization belts around such regions. Moreover, the proposed model can forecast future spatially and age-distributed clusters of infected individuals and provide information to design contention or immunization measures.

Therefore, our main motivation is to present a computational methodology capable of tracking and forecasting the epidemics caused by new emerging pathogens, including SARS-CoV-2, in terms of different geographical regions, sexes, times, and age classes, as well as a calibration procedure that leads to adequate data fitting. This approach in turn allows scenario generation as well as quantitative analysis of public health strategies. See, for example^32^, where an analysis of vaccination delay is performed.

We obtain the geographical distribution of the disease dynamics considering the five NYC boroughs (Manhattan, Bronx, Brooklyn, Queens, and Staten Island) using the census data^33^, the curve of daily new infections^27^, and the corresponding proportions of hospitalizations and deaths depending on age classes, by borough.

### Main findings

After smoothing out the daily curves through $\text {7-}$day moving averages, we estimate the model parameters. The predicted curve by the model for daily new infections shows good agreement with the averaged data curve. Furthermore, the predictions of hospitalizations and deaths match well the reported values based on NYC data and its boroughs.

We observe a dramatic change in the pattern of disease transmission on 19-Mar-2020, identifying the effectiveness of containment measures imposed a week earlier, when a state of emergency was declared and people were asked to stay home. We also observe a considerable decrease in the time-dependent transmission coefficient and the time-dependent basic reproduction rate (obtained via the next-generation matrix technique^34^). We also noticed this phenomenon in the dynamics of the time-dependent transmission coefficients associated with the NYC boroughs.

In the analysis of the datasets, the patterns of daily hospitalizations and deaths changed consistently between the end of February and the end of August, especially the rate of hospitalization, which has decreased systematically since the end of March. To account for this feature, we allow the model rates of hospitalization and death to be time-dependent. This approach produces model predictions in agreement with the datasets.

Short-term forecasts, with calibrated parameters, were also tested in two different situations, namely, during the transmission regime change and after the spread containment. In both cases, the model accurately predicted the observed scenarios.

Moreover, different reopening scenarios were simulated, considering the impacts of reopening the entire NYC, the borough of Staten Island only, or schools only. In all such cases, unless strict social distancing measures were maintained, the model predictions indicate new infection waves that affect the population of the entire city (Figs. 5, 6, 7). Such findings are corroborated by recent news, with new infection waves identified in Europe, New Zealand, and China^35–37^, as well as the reports of COVID-19 spread among a youth population in an overnight camp in Georgia (United States) and in schools in Israel^38,39^.

## Methods

This section presents the epidemiological model and the procedure to estimate the model parameters from the reported data. The estimation of the parameters is performed by minimizing a log-posterior density with a gradient-descent technique. Bootstrap sampling is used to test parameter sensitivity as well as to provide 90% confidence intervals^40^.

### The epidemiological model

The SEIR-type model considered here accounts for disease severity, age range, sex, and geographic distribution of some predefined group or population. For simplicity, we postpone the inclusion of sex and (spatial) location dependence to the end of the present section. A number *n* of age ranges is assumed, each one represented by the superscript $i = 1,\ldots ,n$ and distributed in seven epidemic categories: susceptible ($S^i$), exposed but not yet infective ($E^i$), infective in mild conditions ($I^i_M$), infective in severe condition or hospitalized ($I^i_H$), infective in critical condition or in an intensive care unit (ICU), denoted by $I_I^i$, recovered ($R^i$), and deceased ($D^i$). Following^10^, we assume the following forms are synonyms: in severe condition and hospitalized. The same applies for the forms of in critical conditions and in ICU. Each individual in the first two infective compartments, mildly infective (*M*) and hospitalized (*H*), can recover, die, or develop a more severe disease outcome. The individuals in ICU can only recover or die.

To describe the model, we introduce the following notation: Define the vector

$$\begin{aligned} \mathbf{S} = [S^1,\ldots ,S^n]^T, \end{aligned}$$

where the superscript *T* denotes the transposed vector, and similarly for $\mathbf{E}$, $\mathbf{I} _M$, $\mathbf{I} _H$, $\mathbf{I} _I$, $\mathbf{R}$, and $\mathbf{D}$. Define also the tensor product:

$$\begin{aligned} \mathbf{x} :\mathbf{y} = [x_1y_1,x_2y_2,\ldots ,x_n y_n]^T. \end{aligned}$$

Then, the epidemiological model can be written as:

1 $$\begin{aligned} \dfrac{d\mathbf{S} }{dt}&= - \mathbf{S} :\left( \beta _M \mathbf{I} _M + \beta _H \mathbf{I} _H + \beta _I \mathbf{I} _I\right) \text{, } \end{aligned}$$

2 $$\begin{aligned} \dfrac{d\mathbf{E} }{dt}&= \mathbf{S} :\left( \beta _M \mathbf{I} _M + \beta _H \mathbf{I} _H + \beta _I \mathbf{I} _I\right) - \sigma \mathbf{E} \text{, }\end{aligned}$$

3 $$\begin{aligned} \dfrac{d\mathbf{I} _M}{dt}&= \sigma \mathbf{E} - \left( \nu _M + \mu _M + \gamma _M\right) :\mathbf{I} _M\text{, }\end{aligned}$$

4 $$\begin{aligned} \dfrac{d\mathbf{I} _H}{dt}&= \gamma _M:\mathbf{I} _M -\left( \nu _H + \mu _H + \gamma _H\right) :\mathbf{I} _H\text{, }\end{aligned}$$

5 $$\begin{aligned} \dfrac{d\mathbf{I} _I}{dt}&= \gamma _H:\mathbf{I} _H -\left( \nu _I + \mu _I\right) :\mathbf{I} _I\text{, }\end{aligned}$$

6 $$\begin{aligned} \dfrac{d\mathbf{R} }{dt}&= \nu _M:\mathbf{I} _M + \nu _H:\mathbf{I} _H + \nu _I:\mathbf{I} _I\text{, }\end{aligned}$$

7 $$\begin{aligned} \dfrac{d\mathbf{D} }{dt}&= \mu _M:\mathbf{I} _M + \mu _H:\mathbf{I} _H + \mu _I:\mathbf{I} _I\text{. } \end{aligned}$$

**Figure 1:**
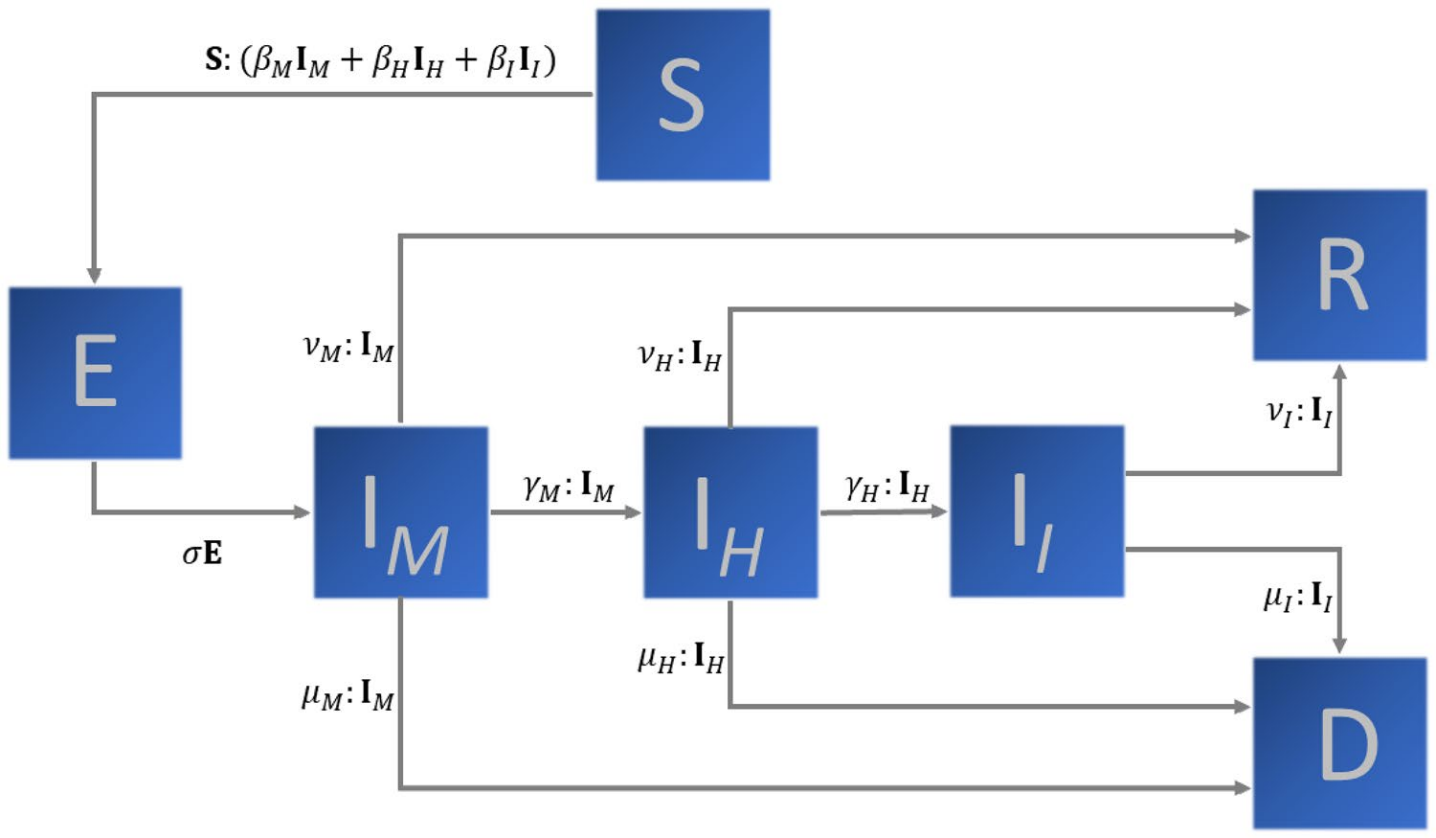
Schematic representation of the epidemiological model described by Eqs. (1)–(7).

A schematic representation of the model in Eqs. (1)–(7) is shown in Fig. 1.

The matrices $\beta _M$, $\beta _H$, and $\beta _I$ contain the transmission parameters for the infective individuals in the mild, hospitalized, and ICU classes, respectively. Such parameters are time-dependent and, if well calibrated, may be used to address the effectiveness of the containment measures, to verify the changes in the transmission pattern, or to track the impact of the suspension of a lockdown. It is important to mention that, depending on the information available, simplifying assumptions on the structure of such matrices must be made. In our study, $\beta _M$, $\beta _H$, and $\beta _I$ assume the following form:

8 $$\begin{aligned} \beta _M = \beta (t)\,\text {B}, ~\beta _H = a\beta _M,~\text{ and }~\beta _I = b\beta _M, \end{aligned}$$

where the symmetric $n\times n$ matrix $\text {B}$ is of the form:

9 $$\begin{aligned} \text {B} = \left[ \begin{array}{ccccc} a_1 &{} a_1b_1 &{} a_1b_2 &{} \cdots &{} a_1b_{n-1}\\ a_1b_1 &{} a_2 &{} a_2b_1 &{} \cdots &{} a_2b_{n-2}\\ \vdots &{} \ddots &{} \ddots &{} \cdots &{} \vdots \\ \vdots &{} \ddots &{} \ddots &{} \ddots &{} \vdots \\ a_1b_{n-1} &{} \cdots &{} a_{n-2}b_2 &{} a_{n-1}b_1 &{} a_n \end{array} \right] , \end{aligned}$$

$\beta (t)$ is a time-dependent scalar parameter that controls the epidemic dynamics, and the parameters *a* and *b* are scale factors between 0 and 1. The matrix $\text {B}$, which describes the mixing between age ranges, depends on $2n-1$ parameters, namely, $a_1,\ldots ,a_n$, containing the observed rates of infections in the *n* age ranges reflecting the observed heterogeneity of the infectiousness of COVID-19 into the model, and $b_1,\ldots ,b_{n-1}$, which will be estimated from the available data. The *n*-dimensional vectors $\nu _M$, $\nu _H$, and $\nu _I$ contain the recovery rates for each age range in the infective classes *M*, *H*, and *I*, respectively. Similarly, $\mu _M$, $\mu _H$, and $\mu _I$ contain the mortality rates for mild, hospitalized, and in ICU infective individuals. The mean time of incubation is $1/\sigma$. The rates $\gamma _M$ and $\gamma _H$ represent the hospitalization and ICU admission, respectively.

The rates of recovery, mortality, hospitalization, and ICU admission are inversely proportional to the corresponding mean times of disease evolution and are directly proportional to the probabilities of moving on to other compartments. All quantities defining such rates are based on the results of references^10,12–14,27–31^.

The time-dependent transmission parameters $\beta _M$, $\beta _H$, and $\beta _I$, as well as the initial number of infective cases, are unknowns and will be estimated from the recorded data of daily new infections. The available census data are used to determine the population size and the proportions of susceptible population on each age range.

Moreover, whenever the data from daily reports of new cases (infections, hospitalizations, and deaths) include different age ranges, the model can be generalized to incorporate such information. In this case, the entries of $\beta _M$ are as follows:

$$\begin{aligned}{}[\beta _M]_{jj} = \beta ^j(t)B_{jj}, ~[\beta _M]_{ij} = \frac{\text {B}_{ij}}{2}\left( \beta ^i(t) + \beta ^j(t)\right) ,~i\not =j, \end{aligned}$$

with $\beta ^j(t)$, $j=1,\ldots ,n$, time-dependent scalar coefficients, and $\text {B}_{ij}$ the entries of the matrix $\text {B}$ defined above. The other transmission parameters are still of the form $\beta _H = a\beta _M$ and $\beta _I = b\beta _M$.

Since for the NYC datasets only the accumulated numbers of infections, hospitalizations and deaths are age-structured, we assume that the daily reported cases are not age-structured. To introduce more realistic death and hospitalization rates, we adjust $\mu _I$ and $\gamma _M$ by appropriate delayed ratios from daily reports. More precisely, if $\overline{\gamma }_M$ and $\overline{\mu }_I$ represent the mean rates of hospitalization and death, respectively, for each age range $i=1,\ldots ,n$, the constant rates $\gamma ^i_M$ and $\mu ^i_I$ are replaced by

10 $$\begin{aligned} \gamma ^i_M\frac{\tilde{I}_H(t)}{\overline{\gamma }_M \tilde{I}_M(t-\tau _M)}\quad \text{ and }\quad \mu ^i_I\frac{\tilde{D}(t)}{\overline{\mu }_I \tilde{I}_H(t-\tau _D)}, \end{aligned}$$

where $\tilde{I}_M$, $\tilde{I}_H$, and $\tilde{D}$ are the time series from the daily reports of new infections, hospitalizations, and deaths, respectively. In addition, $\tau _M$ is the mean time of onset to hospitalization and $\tau _D$ is the mean time from hospitalization to death. We set $\tau _M = 1$, approximating the median value found in^28^, and the parameter $\tau _D$ is set to $\tau _D = 1$, obtained empirically in the numerical tests. Note that we do not consider the curves of daily reports of ICU admissions because these data are not available in the NYC dataset.

#### Including sex in the model

COVID-19 affects male and female individuals differently. Depending on the age range, the case fatality ratio is much larger for male individuals^15,16^. To take sex variance into account in the model, the transmission parameters ($\beta _M$, $\beta _H$, and $\beta _I$) are generalized. The transmission matrix for the mild class assumes the following form:

11 $$\begin{aligned} \beta _M = \left[ \begin{array}{cc} \beta ^F_M &{} \frac{1}{2}(\beta _M^F + \beta _M^M) \\ \frac{1}{2}(\beta _M^F + \beta _M^M) &{} \beta ^M_M \end{array} \right] , \end{aligned}$$

where $\beta _M^F$ and $\beta _M^M$ are the transmission matrices for the age ranges defined above for the female and male individuals, respectively. The transmission matrices $\beta _H$ and $\beta _I$ have the form $\beta _H = a\beta _M$ and $\beta _I = b\beta _M$.

Note that the transmission between sexes is accounted by the mean value of the transmission inside sexes. Intuitively, this means that a female individual who maintains social distancing with other females will also continue such containment measures with male individuals. This assumption also applies to male individuals.

#### Including geographical information

Monitoring and forecasting the disease spread and the effectiveness of containment measures in large regions, such as metropolitan areas, states, and countries, are difficult tasks. Heterogeneous distribution of population and differences in the implementation of social restrictions may lead to quite different disease dynamics among different locations. Moreover, people moving between regions can cause new infection waves. Thus, to account for these aspects, an epidemiological model must include the geographical distribution of the population. A number of approaches have been proposed, and a review on this subject can be found in^20^. In particular, SEIR-type models have been repeatedly used to describe the dynamics of human infectious diseases including geographical information. For example,^23,25^ describe the COVID-19 dynamics in United States counties and in Italy, respectively.

To include the geographical distribution of the population into the model described by Eqs. (1)–(7), we enlarge the transmission matrices. By indexing each site under consideration by $l=1,\ldots ,m$, let $\beta _M^l$, $\beta _H^l$, and $\beta _I^l$ be the corresponding transmission matrices. Then, the transmission matrix for mildly infective individuals in the model becomes:

$$\begin{aligned} \beta _M = \left[ \begin{array}{ccccc} \beta _M^1 &{} c_1 \mathbf{1} &{} c_1 \mathbf{1} &{} \cdots &{} c_{1} \mathbf{1} \\ c_1 \mathbf{1} &{} \beta _M^2 &{} c_2 \mathbf{1} &{} \cdots &{} c_{2} \mathbf{1} \\ \vdots &{} \ddots &{} \ddots &{} \cdots &{} \vdots \\ \vdots &{} \ddots &{} \ddots &{} \ddots &{} \vdots \\ c_{1} \mathbf{1} &{} \cdots &{} c_{m-2} \mathbf{1} &{} c_{m-1} \mathbf{1} &{} \beta _M^m \end{array} \right] , \end{aligned}$$

where $\mathbf{1}$ is the $m\times n$-matrix with all entries equal to one and $c_l = \min _{i,j} [\text {B}^l]_{i,j}$, where $\text {B}^l$ is the matrix defining the transmission matrix $\beta ^l_M$, for the *l*-th location. The transmission matrices for the other infective classes are similar.

This choice for the matrix that represents the mixture of infective populations from different locations helps to simplify the model, considerably reducing the number of unknowns, facilitating calibration. In addition, the model structure is data-driven in the sense that it depends on the current information and thus reflects more precisely the behavior of the population under different containment measures.

When dealing with large areas, such as states and countries, it is important to consider the distance between locations in the model by using exponential, Gaussian, or power law functions^20^. Due to the interconnectedness of NYC and its boroughs, we prefer to estimate the transmission-matrix components from the reported data.

### Estimation procedure

For simplicity, we start by presenting the estimation procedure for the model without sex or geographical dependence. Moreover, the data on new infections, new hospitalizations, and new deaths released by the NYC authorities do not include sex or age ranges. Thus, we use this simpler version of the model, where $\beta ^M = \beta (t)\text {B}$. In addition, to simplify the estimation, the constants *a* and *b*, related to the transmission matrices of hospitalized and in ICU individuals, are empirically set as $a=0.1$ and $b=0.01$, respectively.

To estimate the model parameters from the publicly available curves of daily new infections, we build the so-called posterior distribution relating parameters to data.

We assume that the number of daily new infective cases, denoted by $\mathcal {I}$, is Poisson-distributed with parameter $\sigma \sum _{i=1}^n E^i(t)$. Thus, denoting the vector of model parameters by $\theta$, the logarithm of the likelihood function is

$$\begin{aligned} L(\theta ) \propto \sum _{j=1}^N\left[ \mathcal {I}(t_j). \log \left( \sigma \sum _{i=1}^nE^i(t_j)\right) \sigma \sum _{i=1}^nE^i(t_j) - \log (\mathcal {I}(t_j)!)\right] , \end{aligned}$$

where *N* is the number of the samples in the data and the $\log (\mathcal {I}!)$ is approximated by the Stirling’s formula

$$\begin{aligned} \log (\mathcal {I}!)\approx \frac{1}{2}\log (2\pi \mathcal {I}) + \mathcal {I}\log (\mathcal {I}) - \mathcal {I}. \end{aligned}$$

We also assume that the vector of parameters $\theta$ is Gaussian-distributed with the mean given by some vector of suitably chosen *a priori* parameters, denoted by $\theta _0$, and identity covariance matrix. Thus, the negative of the logarithm of the posterior distribution $lP(\theta |\mathcal {I},\theta _0)$ satisfies

12 $$\begin{aligned} lP(\theta |\mathcal {I},\theta _0) \propto L(\theta |\mathcal {I}) + \frac{\alpha }{2}\Vert \theta -\theta _0\Vert ^2. \end{aligned}$$

The constant $\alpha$ is the so-called regularization parameter in the context of Tikhonov-type regularization methods^1^. The estimated parameters are obtained by minimizing $lP(\theta |\mathcal {I},\theta _0)$.

We estimate the initial proportion of mild infective individual in each age range as follows:

$$\begin{aligned} \mathbf{I} _M(0) = [I^1_M(0),\ldots ,I^n_M(0)]^T \approx I_{M,0}[p_1,\ldots ,p_n]^T, \end{aligned}$$

where $I_{M,0}$ is a scalar and $p_i$ is the population fraction of infective individuals in the *i*-th age range. The latter is estimated from census data. Thus, the vector of the parameters to be estimated assumes the following form:

13 $$\begin{aligned} \theta = [I_{M,0},\beta (t),b_1,\ldots ,b_{n-1}]^T, \end{aligned}$$

where the values of $b_j$, $j~=~1,\ldots ,n-1$, correspond to the entries of the matrix $\text {B}$ in Eq. (9). The time-dependent transmission coefficient $\beta (t)$ also appears in the definition of $\beta _M$.

The estimation of $\theta$ is carried out as follows:

1. Assume that $\beta (t)$ is constant and estimate $\theta$ from the set of daily reports of new infections;
2. Estimate $\beta (t)$ for each $t_j$ in the dataset by minimizing the following functional:
  14 $$\begin{aligned} F\left( \beta (t_{j+1})|\beta (t_j),\theta ,\mathcal {I}(t_{j+1})\right) = \mathcal {I}(t_{j+1}).\log \left( \sigma \sum _{i=1}^nE^i(t_{j+1})\right) - \sigma \sum _{i=1}^nE^i(t_{j+1})\nonumber \\ \quad - \log \left( \mathcal {I}(t_{j+1})!\right) + \alpha \left( \beta (t_{j+1})-\beta (t_{j})\right) ^2,~ \text{ with } j=1,\ldots ,N-1\text{. } \end{aligned}$$

The estimation of the model with geographical information is carried out as follows. Consider the *m*-dimensional time series of daily new reported infections from *m* different locations, where $\mathcal {I}^l$, $l=1,\ldots ,m$, denotes the set of reports for the *l*-th location. For each *l*, let $\theta ^l$ and $\beta ^l(t)$ denote the vector of parameters and the time-dependent transmission coefficient, respectively, of the *l*-th location. We estimate the set of parameters $\Theta = [\theta ^1,\ldots ,\theta ^m]$ and the coefficients $\mathbf {\beta }(t) = [\beta ^1(t),\ldots ,\beta ^m(t)]^T$, by minimizing the log-posterior densities given by:

15 $$\begin{aligned} \sum _{l=1}^m lP(\theta ^l|\mathcal {I}^l,\theta ^l_0) \quad \text{ and } \quad \sum _{l=1}^m F(\beta ^l(t_{j+1})|\beta ^l(t_j),\theta ^l,\mathcal {I}^l(t_{j+1})),\quad j=1,\ldots ,N-1, \end{aligned}$$

where $lP(\theta ^l|\mathcal {I}^l,\theta ^l_0)$ and $F(\beta ^l(t_{j+1})|\beta ^l(t_j),\theta ^l,\mathcal {I}^l(t_{j+1}))$ are given by (12) and (14), respectively. The computational cost of estimating this model varies with the number *m* of the sites considered. Based on the available computational resources, for large m, it may be useful to simplify the model by reducing the number of age ranges or merging the locations into a larger areas, thus reducing the model’s dimension.

We implemented the model’s solution and the estimation procedures in MATLAB (The MathWorks, Inc., Natick, USA). The code is available upon request. The optimization of the posterior density was performed by the general-purpose gradient-based algorithm LSQNONLIN from MATLAB’s Optimization Toolbox.

## Results: estimation, backtesting, and forecasting

We start by evaluating the accuracy of the proposed methodology on fitting real data. Then, we perform back-testing of the estimated results with out-of-sample data for periods of 7 and 20 days. Finally, we perform a forecast analysis under different scenarios.

Our results are based on New York City reports of new infections, hospitalizations, and deaths. This dataset is updated daily and contains information about the disease distribution at the five NYC boroughs with age-structured accumulated numbers^27^.

As the number of daily COVID-19 tests have been increasing in NYC and more effective treatments have been tested and implemented^41^, the rates of new hospitalizations and new deaths have been decreasing relative to the rates of new infections. To account for all these features, the rates used in the model were updated.

### Estimation results

Our initial example set does not yet consider geographical dependence. This information shall be incorporated in “Including geographical information”. The disease dynamics are estimated from the number of daily new infections in the entire NYC area. The data series is from 29-Feb-2020 to 21-Aug-2020 and is obtained from the publicly available data in^27^. This source provides COVID-19 case reports and statistics for NYC and each of its five boroughs. The populations of NYC and of its boroughs are distributed in the 5 age ranges present in the datasets. The population distribution in the age ranges and boroughs is based on the census data publicly available at^33^. The curves of the daily reports of new infections were smoothed-out by a moving average of seven consecutive days. The rates per 100,000 inhabitants were used to define the various model parameters. They include the hospitalization, recovery, and death rates, as well as the vector $\mathbf{a}$ in the definition of the transmission matrices.

**Figure 2:**
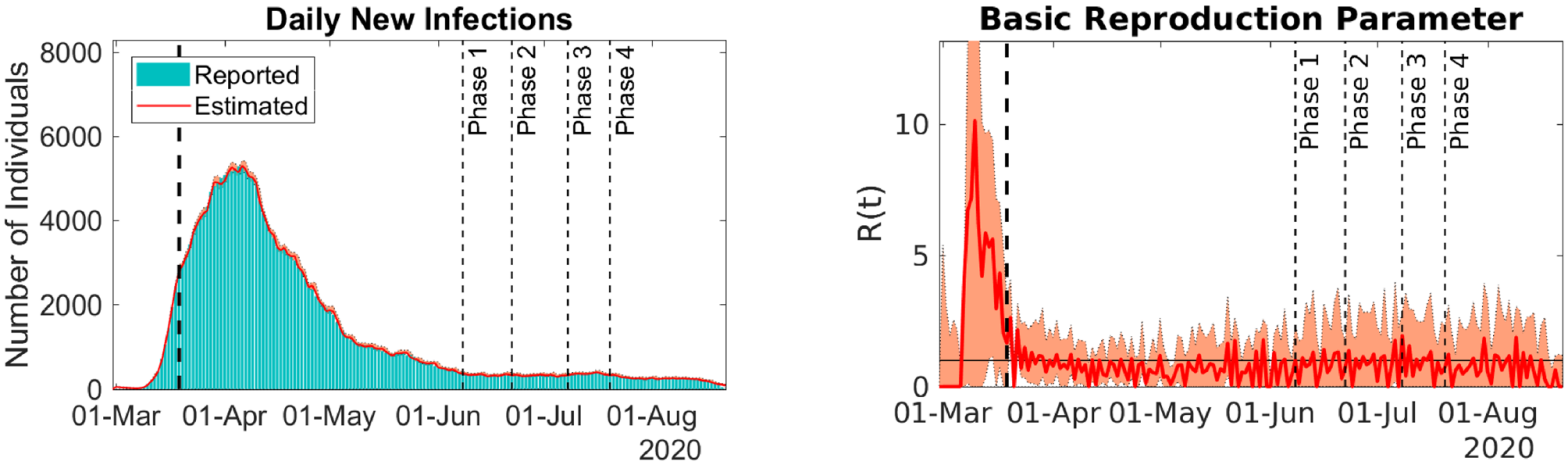
Model predictions and reported daily new infections (left) and time-dependent basic reproduction rate (right). Solid lines represent best-fit predictions, and bars are the 7-day moving average of reported cases. Filled envelopes are the 90% CIs. The vertical dashed lines mark different events in disease dynamics. The first line divides the dataset into uncontained and contained spread, and the remaining lines mark the beginning of reopening phases. NYC data. Figure generated with MATLAB R2019b (mathworks.com).

After preliminary calibration tests, two disease evolution regimes were clearly identified. In the first regime, the number of infective individuals increased exponentially, and in the second regime, the spread was considerably reduced due to the containment measures imposed by the state of emergency declared on 12-Mar-2020. The effect of such intervention was clearly observed by the change in the time-dependent transmission coefficient $\beta (t)$ on 19-Mar-2020 (Fig. 2).

To capture possible regime changes, such as the different age-range mixing, we divided the time-series into two parts, namely, the data before and after 19-Mar-2020. For these two time series subsets, we estimate the vector of parameters $\theta$ and the time-dependent $\beta$. Note that for the second dataset, after 19-Mar-2020, we do not estimate the initial infective population. The estimated parameters and the corresponding 90% confidence intervals (CIs) can be found in Table 1. Such intervals were obtained by excluding the 5% largest and smallest values generated by 200 bootstrap samples^40^. Figure 2 presents the comparison between the reported and model predicted curves for daily new infections. The time evolution of the basic reproduction rate can be found in Fig. 2. Table 2 depicts the predicted and reported number of infections, hospitalizations, and deaths for 21-Aug-2020 with 90% CIs.

**Table 1:** Best fit and 90% CIs of the model parameters obtained from the time-series subsets of daily reported new infections in NYC.

	Subset 1	Subset 2
$I_{M,0}$	2.0 (2.0–2.0)	–
$\beta$	8.29 (8.15–8.35)	2.81 (2.71–3.20)
$b_1$	0.88 (0.87–0.89)	0.51 (0.50–0.81)
$b_2$	0.81 (0.79–0.81)	0.24 (0.01–0.31)
$b_3$	0.77 (0.75–0.78)	0.19 (0–0.53)
$b_4$	0.52 (0.32–0.58)	0.17 (0.11–0.85)

In Table 1, the estimated values of $\beta$ were far from each other in both periods, indicating the aforementioned change in the transmission regime. For the vector $\mathbf{b}$, after containment, the estimated values decrease consistently, indicating that the interaction between age ranges was also significantly reduced. All these results show that disease spread was contained after 19-Mar-2020. However, as we shall see later on, new infection waves can occur if containment measures are relaxed.

**Table 2:** Model predictions with 90% CIs (top rows) and reported numbers (bottom rows) of accumulated infections, hospitalization, and deaths for NYC on 21-Aug-2020, by age range and sex.

	Infections	Hospitalizations	Deaths
**Age**			
0–17	7443 (6904–10050)	610 (566–822)	11 (10–15)
	7252	623	12
18–44	88,526 (82,183–119,054)	9175 (8520–12,321)	716 (665–966)
	86,413	9297	728
45–64	83,391 (77,466–111,818)	18,712 (17,388–25,054)	4217 (3923–5675)
	81,125	18,958	4268
65–74	26,131 (24,271–35,046)	12,856 (11,947–17,216)	4889 (4557–6587)
	27,221	12,434	4707
75+	23,098 (21,456–30,947)	16,712 (15,534–22,356)	10,070 (9408–13,569)
	25,653	15,568	9298
Total	228,588 (212,280–306,916)	58,065 (53,954–77,769)	19,902 (18,564–26,812)
	228,144	56,882	19,014
**Sex**			
Female	114,649 (106,465–153,971)	24,215 (22,499–32,443)	7212 (6725–9719)
	111,713	24,934	7638
Male	113,939 (105,815–152,945)	33,850 (31,455–45,326)	12,690 (11,839–17,094)
	116,239	31,936	11,373

Figure 2 shows the adherence of the calibrated model predictions to the 7-day moving average of the reported number of new infections. The hospitalization and death rates were evaluated using the ratios of the reported data defined in Eq. (10). The proportions of ICU admissions by age were obtained in^14^ and were adjusted according to the proportions of deaths available in^27^. The model accuracy is also illustrated in Table 2, showing that the model predictions for the accumulated numbers of infections, hospitalizations, and deaths, for each age range and sex, are close to the reported values. We also evaluate the relative error of the best fit model infections. It has a median value of $6.5 \times 10^{-4}$ and a 90% CI of $8.9\times 10^{-6}$ to 0.09.

It is clearly observed from Fig. 2 that the time-dependent basic reproduction rate *R*(*t*) values can be classified into two different categories. Prior to 19-Mar-2020, *R*(*t*) has large values, indicating that the disease was spreading uncontrollably. After 19-Mar-2020, the transmission parameter value drops to approximately one, indicating the control of transmission by containment measures imposed from 12-Mar-2020 onwards. Note that the large values for the basic reproduction rate in the first part of the series, i.e., prior to 19-Mar-2020, may be caused by an accumulation of reports in the beginning of the outbreak. Such accumulation can be related, for example, to the difficulties faced by the health authorities in setting up an appropriate diagnosis protocol.

The adherence of the calibrated model predictions to reported data, the accuracy in the number of hospitalizations and deaths, as well as the behavior of the calibrated parameters indicating the effect of disease containment measures for the NYC data show that our proposed model captures the COVID-19 dynamics in NYC well. Therefore, it is useful to track the spread dynamics, allowing to assess the effects of, for example, travel quarantine, social distancing, and reopening strategies. If infection curves for different age ranges are available, it is possible to use the present model to track aspects such as the effects of reopening schools, universities, or parks and public gardens since these spaces are usually frequented by people of well-defined age ranges. Thus, it is easier to track the disease spread dynamics more accurately, allowing the decision of whether additional reopening or further restrictions can be implemented.

### Including geographical information

Let us consider the epidemiological dynamics of the five boroughs of NYC, namely, Queens, Manhattan, Staten Island, Brooklyn, and the Bronx. The datasets for the five boroughs were downloaded from^27^ on 02-July-2020.

**Figure 3:**
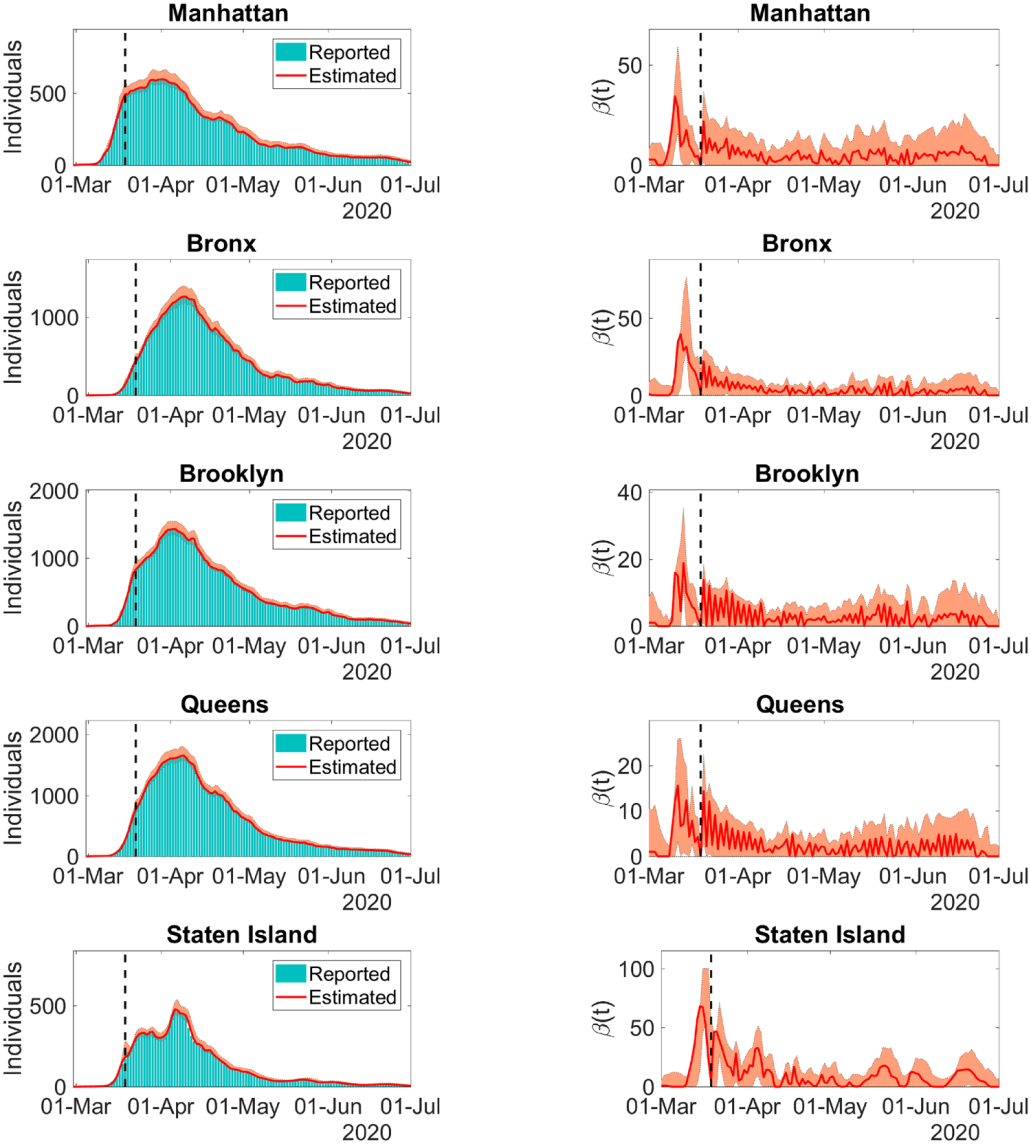
Left column: Model predictions and reported daily new infections for each NYC borough. Right column: Time-dependent transmission parameter estimated for the NYC each boroughs. Solid lines represent the model best fit predictions, and bars depict the 7-day moving average of the number of reported cases. Dashed lines divides the spread regime into uncontained and contained. The filled envelopes are the 90% CIs. The time series is from 29-Feb-2020 to 01-July-2020. Figure generated with MATLAB R2019b (mathworks.com).

The calibrated model predictions of daily new infections and the corresponding time-dependent transmission coefficients can be found in Fig. 3.

**Table 3:** Model predictions and reported accumulated infections, hospitalizations, and deaths for the boroughs of NYC on 01-July-2020.

Borough	Infections	Hospitalizations	Deaths
Manhattan	26,886 (26,793–28,994)	8560 (8540–9269)	2826 (2805–3052)
	26,809	8081	2448
Bronx	47,870 (46,565–51,577)	13,618 (13,486–15,226)	4882 (4867–5629)
	47,691	12,233	3830
Brooklyn	59,287 (58,357–63,889)	14,062 (13,900–15,177)	4855 (4741–5260)
	59,037	15,351	5495
Queens	64,962 (63,939–70,092)	15,571 (15,318–16,786)	4917 (4829–5270)
	64,797	16,977	5839
Staten Island	14,131 (14,131–15,421)	2287 (2287–2523)	840 (840–927)
	13,961	2351	872

Top rows represent predictions and bottom rows are reported cases. Inside the parentheses are the 90% CIs.

Figure 3 shows the model predictions adherence to the curves of reported daily new infections. The high accuracy of the model is also evidenced by the comparison between the reports and predictions of the accumulated number of total infections, hospitalizations, and deaths for 01-July-2020 in Table 3.

The behavior of the time-dependent transmission coefficient for each borough is similar to the basic reproduction rate *R*(*t*) in the previous example. This behavior is expected since transmission containment measures were undertaken since 12-Mar-2020 in the entire NYC.

This example demonstrates the ability of the present model to detect disease transmission patterns in different locations at the same moment. The model also accounts for the interaction between individuals of different age ranges, sexes, and locations. Using such features, it is possible to track the implications of reopening, the necessity of additional containment measures, or modification of the design of vaccination strategies. Such broad applications could not be achieved via simpler models. The median and 90% CI of the relative error of the best fit model infections are $6.7\times 10^{-4}$ and $2.0\times 10^{-5}$ to 0.03, respectively.

### Backtesting

To test the short-term forecast capabilities of the model, we consider two different periods of the COVID-19 outbreak in NYC.

#### Uncontained spread

We calibrate the parameters with data from reports on new infections in the period from 29-Feb-2020 to 19-Mar-2020, and we produce a seven-day forecast starting on 20-Mar-2020. This forecasted period is of particular interest since on 19-Mar-2020, the disease spread pattern changed considerably due to the containment measures undertaken 7 days earlier, as a consequence of the state of emergency declared on 12-Mar-2020. To generate the predictions, we assume that $\beta (t)$ is constant for dates *t* after 19-Mar-2020 and that it takes the same value as that estimated on 19-Mar-2020.

**Figure 4:**
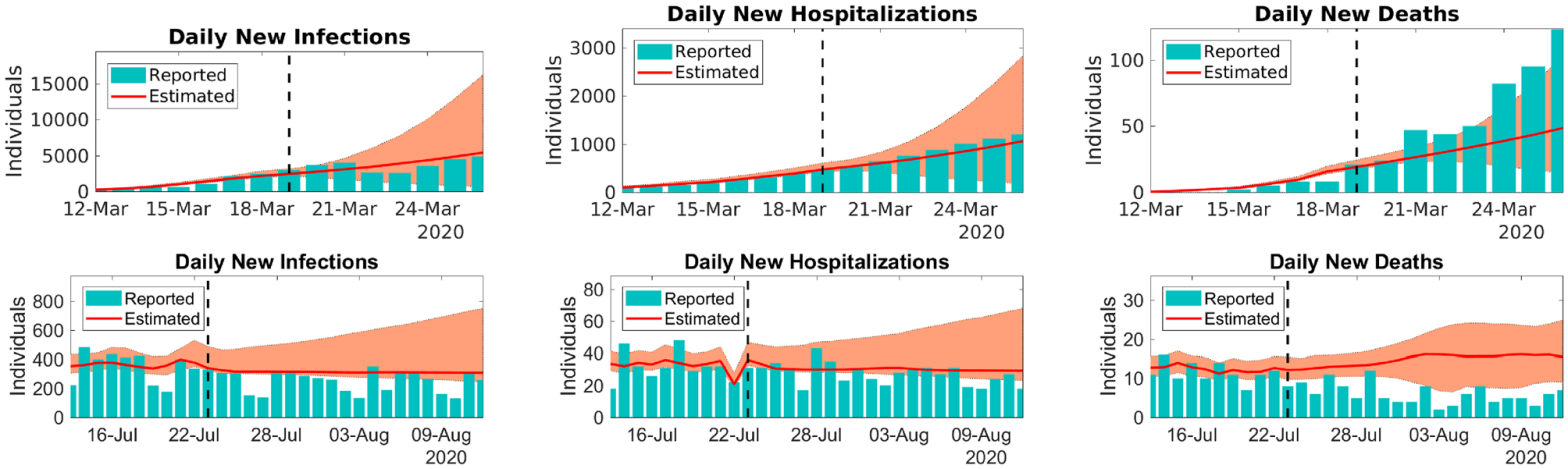
Predictions for the daily reported new infections (left), new hospitalizations (center), and new deaths (right) for the periods 19 to 24-Mar-2020 (top row) and 24-July-2020 to 12-Aug-2020 (botton row). The solid lines represent the model predictions with best fit and the bars that depict the reported NYC data. The model forecasts are presented on the right side of the dashed lines. The filled envelopes are the 90% CIs. Figure generated with MATLAB R2019b (mathworks.com).

Figure 4 presents a comparison between the forecasted curves and the reported data. Although the parameters were estimated with information based on uncontrolled disease spread, the 7-day ahead forecast for daily new infections and new hospitalizations starting on 20-Mar-2020 show satisfactory accuracy. The accumulated number of infections, hospitalizations, and deaths for the forecasted period can be found in Table 4.

#### Contained spread

We now generate a forecast for the period 24-July-2020 to 12-Aug-2020. For dates *t* after 23-July-2020, the time-dependent values of the transmission coefficient are given by the mean of values for the period from 13 to 23-July-2020.

Figure 4 presents a comparison between the predicted and the reported data for the forecasted period. Table 4 presents the predicted and the reported values of accumulated infections, hospitalizations, and deaths from 24-July-2020 to 12-Aug-2020.

**Table 4:** Model predictions and reported accumulated infections, hospitalizations, and deaths for the periods 19 to 26-Mar-2020 and 24-July to 12-Aug-2020.

Period	Infections	Hospitalizations	Deaths
19-Mar to 26-Mar	27,838 (8544–61,140)	5456 (2181–10,819)	245 (143–390)
	25,876	6141	465
24-July to 12-Aug	6262 (5581–11,754)	602 (534–1057)	297 (193–393)
	6654	736	147

Top rows represent predictions, while bottom rows are the reported cases. Between parentheses are the 90% CIs.

As Fig. 4 and Table 4 show, the model predictions of infections, hospitalizations, and deaths once again show satisfactory accuracy. These results are explained by the model ability to incorporate the disease dynamics through the time-dependent parameters. Note that in these examples, the rates of hospitalizations and deaths are evaluated using appropriate ratios of reported data, as defined previously, until the last day of estimation. In the forecasted period, we repeat the rates for the days 19-Mar-2020 and 23-July-2020, respectively, for the corresponding data ranges studied.

### Reopening scenarios

We now apply the calibrated models from the previous sections to a number of plausible scenarios, such as the reopening of the entire NYC region, reopening of schools, and reopening of only one single borough, which we chose to be Staten Island. The predictions for these scenarios will illustrate the predictive capability of our model.

#### The entire NYC

The aim of this example is to present possible scenarios for the COVID-19 epidemic for long periods without an effective vaccine or appropriate treatment. We consider two different scenarios. In the first scenario, the transmission parameters stay at the level of strict containment, as was observed in the period from 04 to 14-June-2020. Thus, for any date *t* after 14-June-2020, the transmission coefficient $\beta (t)$ is set as the mean for the estimated values of $\beta (t)$ in the period from 04 to 14-June-2020, i.e., $\beta (t) = 1.77$ with 90% CI 0.27–4.69. In the second case, we simulate a controlled reopening by allowing the coefficient $\beta (t)$ to reach twice the values obtained in the previous case, i.e., $\beta (t) = 3.54$ (0.53–9.38). However, if we impose the condition that whenever the number of daily new infections reaches 1000 cases, containment measures are undertaken, forcing $\beta (t)$ to return to lower levels until it reaches the value 1.77 (0.27–4.69). On the other hand, after undertaking containment measures, if the number of daily new infections is below 200 cases, we permit reopening, and $\beta (t)$ may increase again until it reaches 3.54 (0.53–9.38).

**Figure 5:**
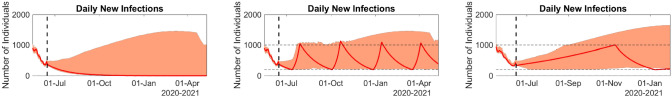
Model predictions of daily new infections for reopening scenarios. Left: containment measures are maintained during the whole period. Center: Reopening is allowed until the daily number of new infections reaches 1000 or if it is below 200 (horizontal dashed lines). Right: Reopening schools. The filled envelopes represent the 90% CIs. Figure generated with MATLAB R2019b (mathworks.com).

Figure 5 (left and center panels) present the curves of daily new infections for the aforementioned scenarios. Before 14-June-2020, we have the estimated curves. The forecasting is carried out from 15-June-2020 to 11-May-2021. According to this example, the reopening without an effective vaccine or the achievement of herd immunity may give rise to new infection waves, even when the number of daily new cases is relatively low.

#### Reopening schools

To simulate a controlled reopening of schools from 15-June-2020, we use the set of parameters estimated after 19-Mar-2020. To artificially increase the interaction between school-age individuals, the entries of the transmission matrix $\beta _M$ associated with the mildly infective individuals in the age range 0 to 17 years old are multiplied by 2.5. In addition, the time-dependent transmission coefficient is set to 1.25 times the mean of the estimated values for the period 06 to 15-June-2020. For this specific age range, the transmission parameter values are similar to those obtained for the period before 19-Mar-2020. This result is expected since controlling the mixing in youth population at schools is difficult. Indeed, recent news indicates that the COVID-19 transmission rate among people under 19 years old is similar to that in other age ranges^38,39^.

Figure 5 (right panel) presents model predictions of this “safe” reopening. Note that whenever the number of daily new infections reaches 1000 cases, containment measures are imposed again. Reopening occurs if the number of daily new cases falls below 200.

Therefore, even in an idealized situation, reopening schools may cause new infection waves among the entire population. Thus, monitoring transmission dynamics is of fundamental importance to set the appropriate time for relaxing or tightening containment measures. In Israel, the recent reopening of schools caused a secondary wave of new infections that forced the adoption of new containment measures^39^.

#### Reopening Staten island

Two different scenarios are considered in this example: reopening Staten Island with and without restrictive measures. In the first scenario, containment measures are slightly relaxed, without allowing people from different age ranges and boroughs to interact. Quantitatively, we keep the same parameter values estimated in the period 19-Mar-2020 onwards. The only change is in the transmission coefficient for Staten Island, which is set to twice the mean of the corresponding estimated values of $\beta (t)$ for the period 22-June-2020 to 01-July-2020, for dates *t* after 01-July-2020. During the forecasted period (02-Jul-2020 to 29-Oct-2020), the transmission coefficients for the other boroughs are kept equal to the mean of the estimated values for the period 22-June-2020 to 01-July-2020.

In the second scenario, people from different boroughs and age ranges are allowed to interact, keeping some social distancing and simple containment measures. However, in Staten Island, only simple containment measures are undertaken. In other words, the time-independent transmission parameters assume the same values estimated in the period 29-Feb-2020 to 19-Mar-2020. In addition, we allow the time-dependent transmission coefficient for Staten Island to reach twice the mean of the estimated values for the period 22-June-2020 to 01-July-2020, for dates *t* after 01-July-2020. Again, after 01-July-2020, the transmission coefficients for the other boroughs are kept equal to the mean of the estimated values for the period 22-June-2020 to 01-July-2020. Whenever the number of daily new infections reaches 1000 in NYC, containment measures are undertaken again. Therefore, the time-independent transmission parameters are brought back to the same values of the period after 19-Mar-2020, and the transmission coefficient for Staten Island is reset to the mean of the corresponding estimated values for the period 22-June-2020 to 01-July-2020. Reopening reoccurs whenever the number of daily new infections in NYC is below 100 reports.

**Figure 6:**
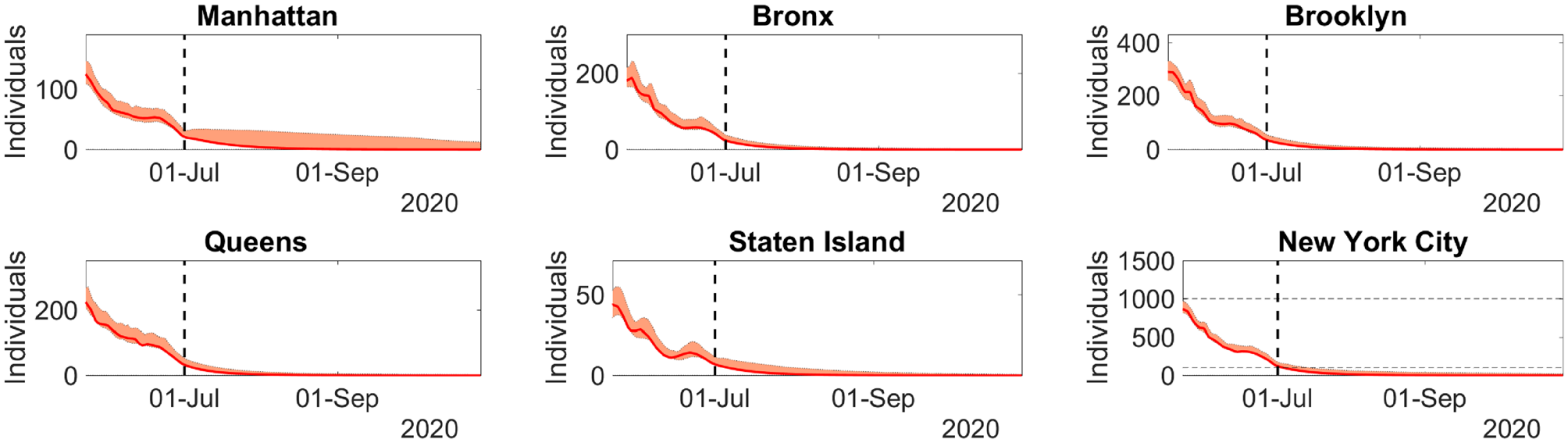
Model predictions of daily new infections for the period 02-July-2020 to 29-Oct-2020 in NYC, when lockdown is slightly lifted, but keeping strict containment measures. The filled envelopes represent the 90% CIs. Figure generated with MATLAB R2019b (mathworks.com).

**Figure 7:**
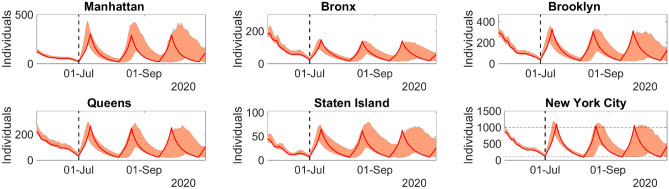
Model predictions of daily new infections for the period 02-July-2020 to 29-Oct-2020 in NYC, when lockdown is lifted, with light containment measures. The filled envelopes represent the 90% CIs. Figure generated with MATLAB R2019b (mathworks.com).

Figures 6 and 7 present the curves of daily new infections for the first and second scenarios, respectively. They show the evolution for each borough and for the entire NYC. In the first case, doubling the mean of the transmission coefficient alone is not sufficient to cause secondary waves of infection. In other words, if restriction of movement between boroughs is maintained and the interaction between age ranges remains contained through strict social distancing measures, infection will not return and the disease outbreak will die out. On the other hand, reopening a borough and allowing people of different ages and from different boroughs to interact, even while keeping some light containment and social distancing measures, can cause new waves of infection for large periods.

## Discussion

We use a 7-day moving average to perform smoothing of the data. After calibration, the model predictions were adherent to the data of daily new infections and accurately predicted the number of daily new hospitalizations and deaths. The adjustment of the rates of hospitalization and death using appropriate ratios of reported data contributed to improving model predictions. While backtesting, forecasts for few days ahead under different contexts were found to be accurate. The model accurately identified the effects of the lockdown undertaken in NYC after 12-Mar-2020. A considerable change in the values of the transmission rates was observed. This change flattened the curve and kept the number of daily new infections low, as shown in^27^.

The rates of hospitalizations and deaths were lower by the end of August than in earlier periods of the COVID-19 outbreak in NYC. This phenomenon may be caused by the changes in disease virulence or in the protocols used to address COVID-19, such as smaller onset to hospitalization mean time, more precise COVID-19 diagnosis, or the introduction of more effective treatments^41^. Moreover, in NYC, the number of COVID-19 daily tests has been increasing consistently since the beginning of the outbreak in the end of February 2020. To account for such changes in the aforementioned rates, the corresponding model parameters were adjusted using the ratios of reported data, incorporating this feature. This considerably increased the accuracy of the model predictions of hospitalizations and deaths, as shown by the results (Fig. 2 and Table 1).

With regard to reopening strategies, some simulated scenarios generated with calibrated parameters indicate that there is no completely safe method for reopening schools, boroughs, or the entire city unless people respect strict social distancing protocols, avoiding direct personal contact. As model predictions show, even when only a borough or only schools are reopened, new infection waves may occur, forcing public authorities to establish containment measures again. As long as infective individuals are present in a population, there is the risk of new infection waves in reopening strategies since it is not possible to guarantee that everyone will respect the containment protocols. This phenomenon was recently reported^37,39^. Thus, reopening must be undertaken with strict control of disease transmission, while applying massive testing and enforcing social distancing measures.

Although the role of children and teenagers in COVID-19 spread is still unknown, some recent events of disease spread among a youth population in an overnight camp in Georgia^38^ and in schools in Israel^39^ indicate that even though most individuals in this age group present mild symptoms, they can infect other people. Therefore, reopening schools also represents a risk of new infection waves.

Even New Zealand and China, which successfully contained COVID-19 spread and had long periods without registering community transmission, are now facing new cases^35,36^. All these reports corroborate our model predictions, suggesting that without strict control of COVID-19 through social distancing or after massive and effective vaccination, a completely safe reopening may be impossible.

It is important to mention that the present model is also suited to simulate and analyze vaccination strategies because it addresses the dependence of the disease outbreak on age and spatial distribution. This possibility will be addressed in a forthcoming article.

### Tracking the reopening of NYC

On May 2020, the State of New York initiated a four-phase reopening program. NYC entered the fourth phase on 20-July-2020. By 22-Aug-2020, schools and shopping malls were still closed, yet public transportation and a number of economic activities were already operational^42,43^. Strict social distancing measures were still enforced, and public authorities were closely following their observance. This approach kept the number of daily new infections stable.

Figure 2 shows the COVID-19 situation in NYC until 21-Aug-2020. The panel presents the comparison of model predictions and reports of daily new infections, as well as the time-dependent basic reproduction rate *R*(*t*). We observe that since 19-Mar-2020, *R*(*t*) stays near one, meaning that disease transmission is under control, but not eradicated, and that new infection waves may still occur. Even after reaching the fourth phase of controlled reopening, NYC authorities managed to keep transmission under control through social distancing measures by limiting the operational capacity of numerous services, disinfecting public transportation, and many other practices.

Note that even if COVID-19 is eradicated in NYC, as long as no effective vaccine is ready for worldwide use, containment measures inside NYC must be continued to avoid new outbreaks caused by the reintroduction of the disease from abroad.

## Concluding remarks

SEIR-type models have been proposed by a number of authors to predict qualitative aspects of the dynamics of infectious diseases in general and of the COVID-19 pandemic in particular. See^44^ for a recent account of the SIR models and their connections to other models. However, to address the elusive aspects of the complex human interactions within the terrain, we think that it is necessary to forego parsimonious models. Functional and high-dimensional models have been used in a number of areas ranging from financial mathematics to population dynamics^45,46^. They are directly connected to the mathematical theory of inverse problems^1,2,22,23^.

The novelty of the work includes incorporating several aspects of the COVID-19 outbreak that have received little attention in the recent literature and provide sufficient flexibility for excellent adherence to real data. We consider time-dependent rates of transmission, hospitalization, and deaths, as well as the disease age- and sex-dependent severity and transmission, while taking into account the spatial distribution of the population.

The model was extensively tested with real data from NYC and its boroughs. After calibration, the model predictions matched the curves of daily new infections, and the model provided accurate predictions for the number of daily hospitalizations and deaths. The relative error of the best-fit model infections was of the order of less than 1% in all cases, indicating the excellent fitting of the models. The model also correctly detected the change in the transmission pattern on 19-Mar-2020 caused by containment measures undertaken on 12-Mar-2020. Moreover, it illustrated the stabilization of the time-dependent basic reproduction rate around the value 1 in NYC.

Concerning the prediction of new infections, the model was also evaluated while using real data and calibrated parameters. It generated accurate results under controlled and uncontrolled transmission contexts. Moreover, different scenarios such as reopening of schools and of an entire borough of NYC were examined. In both cases, transmission rates increased considerably, demanding new containment measures. In other words, without reaching herd immunity or complete disease eradication, we will always face the risk of new infection waves.

Our proposed model is sufficiently general to track transmission dynamics with dependence on age range, sex, and spatial distribution, while evaluating the disease impact on the population. Thus, it is a powerful tool to evaluate scenarios and build proper vaccination strategies.

## Data Availability

The datasets analysed are available in the NYC Health website COVID-19: Data https://www1.nyc.gov/site/doh/covid/covid-19-data.page.

## Code Availability

The code that was used to obtain the findings of the present study is available from the corresponding author upon reasonable request.
